# Epstein-Barr Virus-Encoded Latent Membrane Protein 1 Impairs G2 Checkpoint in Human Nasopharyngeal Epithelial Cells through Defective Chk1 Activation

**DOI:** 10.1371/journal.pone.0039095

**Published:** 2012-06-25

**Authors:** Wen Deng, Pei Shin Pang, Chi Man Tsang, Pok Man Hau, Yim Ling Yip, Annie L. M. Cheung, Sai Wah Tsao

**Affiliations:** Department of Anatomy, Li Ka Shing Faculty of Medicine, The University of Hong Kong, Hong Kong, China; Karolinska Institutet, Sweden

## Abstract

Nasopharyngeal carcinoma (NPC) is a common cancer in Southeast Asia, particularly in southern regions of China. EBV infection is closely associated with NPC and has long been postulated to play an etiological role in the development of NPC. However, the role of EBV in malignant transformation of nasopharyngeal epithelial cells remains enigmatic. The current hypothesis of NPC development is that premalignant nasopharyngeal epithelial cells harboring genetic alterations support EBV infection and expression of EBV genes induces further genomic instability to facilitate the development of NPC. The latent membrane protein 1 (LMP1) is a well-documented EBV-encoded oncogene. The involvement of LMP1 in human epithelial malignancies has been implicated, but the mechanisms of oncogenic actions of LMP1, particularly in nasopharyngeal cells, are unclear. Here we observed that LMP1 expression in nasopharyngeal epithelial cells impaired G2 checkpoint, leading to formation of unrepaired chromatid breaks in metaphases after γ-ray irradiation. We further found that defective Chk1 activation was involved in the induction of G2 checkpoint defect in LMP1-expressing nasopharyngeal epithelial cells. Impairment of G2 checkpoint could result in loss of the acentrically broken chromatids and propagation of broken centric chromatids in daughter cells exiting mitosis, which facilitates chromosome instability. Our findings suggest that LMP1 expression facilitates genomic instability in cells under genotoxic stress. Elucidation of the mechanisms involved in LMP1-induced genomic instability in nasopharyngeal epithelial cells will shed lights on the understanding of role of EBV infection in NPC development.

## Introduction

Epstein-Barr virus (EBV) infects over 95% of adult population in the world. EBV readily infects infiltrating B-cells in the epithelium of the naso- and oro-pharyngeal mucosa of the upper respiratory tract [1]. EBV persists in a lifelong latent infection state in memory B-cells of most healthy individuals. Disruption of this latency leads to the production of infectious virions that can infect permissive epithelial cells and other B-cells. EBV infection is associated with human malignancies. Among all EBV-associated epithelial malignancies, the association between EBV infection and nasopharyngeal carcinoma (NPC) is the strongest [1], [2].

NPC is a common cancer in Southeast Asia, particularly in southern regions of China including Hong Kong. The incidence of NPC in ethnic Chinese living in southern China, including Hong Kong, is ranging 50 to 100 folds higher than non-Chinese populations in North America and Europe [1], [3]. In undifferentiated NPC, which is the typical histopathological type of NPC in southern China, EBV could be detected in most, if not all, NPC cells [1]. EBV infection has been postulated to be a crucial etiological factor in NPC pathogenesis, yet the underlying oncogenic mechanisms of EBV in NPC remain elusive. Deletions in chromosomes 3p and 9p could be detected in dysplastic lesions and histologically normal nasopharyngeal epithelium of southern Chinese prior to EBV infection [4], [5]. This leads to the hypothesis that genetically altered premalignant nasopharyngeal epithelial cells support EBV infection, and expansion of a specific EBV-infected clone of premalignant nasopharyngeal epithelial cells with the expression of lytic and latent genes of EBV drives further genomic instability in the EBV-infected nasopharyngeal epithelial cells, eventually leading to tumorigenic transformation.

Latent membrane protein 1 (LMP1) is a well-documented EBV-encoded oncogene. LMP1 expression resulted in tumorigenic transformation of rodent fibroblast cells [6]. Transgenic mice expressing LMP1 developed B cell lymphoma [7]. LMP1 is commonly expressed in Hodgkin’s lymphoma and nasal lymphoma [1]. LMP1 expression could be detected in preinvasive NPC lesions (NPC *in situ*) infected with EBV [8]. LMP1 expression facilitates immortalization of nasopharyngeal epithelial cells by telomerase [9]. All these observations support an important role of LMP1 in the early pathogenesis of NPC. Furthermore, LMP1 modulates multiple cell signaling pathways through activation of nuclear factor-kappa-B (NF-kB), Janus-activated kinase/signal transducer and activator of transcription (JAK/STAT), mitogen-activated protein kinase (MAPK), protein kinase B (PKB) and other signaling pathways to induce survival, anti-apoptosis and invasive properties in EBV-infected cells [1], [10].

The G2 checkpoint is essential for cell survival and maintenance of genomic stability [11], [12]. It delays cell cycle progression from G2 to M phase to provide time for correction of DNA damage or replication errors. Defective G2 checkpoint allows cells that carry chromosome aberrations to exit G2 and enter mitosis [13], leading to genomic instability which facilitates carcinogenesis. The impact of LMP1 on G2 checkpoint in nasopharyngeal epithelial cells has not been previously examined. In this study, we found that LMP1 impaired G2 checkpoint in nasopharyngeal epithelial cells, leading to formation of unrepaired chromatid-type aberrations in metaphase cells. We further found that defective Chk1 activation was responsible for the induction of defect in G2 checkpoint in LMP1-expressing nasopharyngeal epithelial cells.

## Results

### Stable Expression of LMP1 Impairs G2 Checkpoint Function

**Figure 1:**
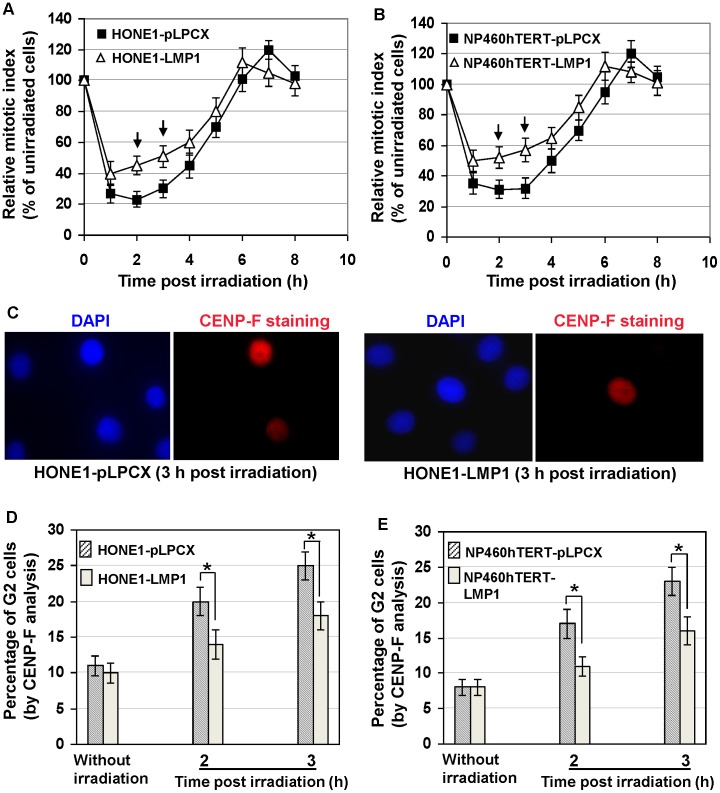
G2 checkpoint defect induced by LMP1. A and B, Relative mitotic index at different time points after γ-ray irradiation (relative percentage of mitotic index as compared with unirradiated cells). Error bars represent standard deviations. Arrows indicated the relative mitotic indices in LMP1-expressing cells significantly higher (*P*<0.05) than empty vector-infected control cells. C, Examples of G2 cells identified by pan-nuclear CENP-F staining. DNA was stained by DAPI. D and E, Quantification of percentage of G2 cells with or without γ-ray irradiation. Error bars represent standard deviations. Stars indicated significant differences (*P*<0.05) between the indicated datum pairs.

To study the effect of LMP1 on G2 checkpoint function, we stably expressed LMP1 in HONE1 (an EBV-negative nasopharyngeal carcinoma cell line) [14] and NP460hTERT cells (an hTERT-immortalized nasopharyngeal epithelial cell line) [15]. The cells were infected with retroviral vectors expressing 2117-LMP1 (pLPCX-2117LMP1) or empty vectors (pLPCX), and selected with puromycin for 6 days. The puromycin-resistant cells were expanded for further functional studies on G2 checkpoint. The 2117- LMP1 is a representative NPC-derived LMP1 variant present in 86% of NPC patients in Hong Kong, which is an endemic area of NPC [16]. Cells with intact G2 checkpoint will be arrested at G2 after DNA damage, while G2-defective cells will continue to exit from G2, enter mitosis and progress into the next G1 phase. Hence, the function of G2 checkpoint could be readily monitored by the decrease in percentage of mitotic cells (mitotic index) several hours after γ-ray irradiation as compared with mitotic index of unirradiated control cells (represented as relative mitotic index) [17]–[19]. After γ-ray irradiation which induces DNA damage, cells with defective G2 checkpoint will have a relatively higher mitotic index compared with cells with intact G2 checkpoint. The mitotic cells could be readily identified by distinguishable chromosome spreading using cytogenetic methods. We found that LMP1-expressing cells exhibited impaired G2 checkpoint function, as demonstrated by the significantly (p<0.05) higher relative mitotic indices compared with empty vector-infected cells 2–3 h after 0.5 Gy γ-ray irradiation (Figures 1A and 1B).

The nuclear CENP-F staining, which is a specific marker for G2 cells [19]–[21], was used to identify G2 cells 2–3 h after 0.5 Gy γ-ray irradiation (Figure 1C). Mitotic cells could be excluded by discrete centromeric CENP-F staining and condensed chromatin (Figure S1). As shown in Figures 1D and 1E, the percentages of G2 cells in LMP1-expressing cells in the absence of γ-ray irradiation were not significantly different from empty vector-infected cells. In contrast, 2–3 h after 0.5 Gy γ-ray irradiation, significantly lower percentages of G2 cells were observed in LMP1-expressing cells compared with empty vector-infected cells (Figures 1D and 1E. This further confirmed that LMP1-expressing cells were impaired in G2 checkpoint.

### LMP1-expressing Cells Released from G2 Checkpoint Contained Increased Frequencies of Chromatid Breaks Compared with Control Cells

**Figure 2:**
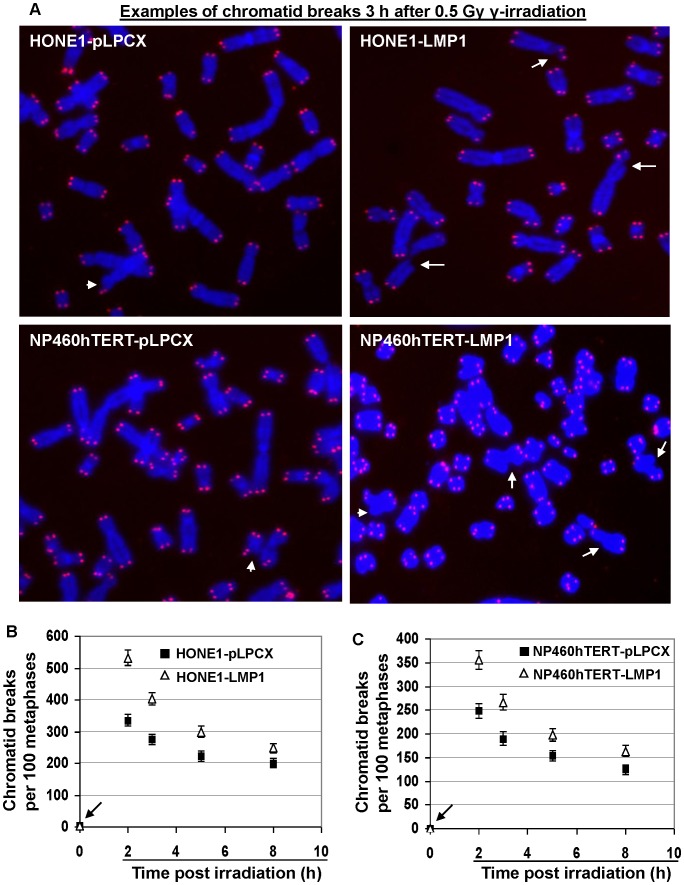
Frequencies of chromatid breaks in LMP1-expressing and control cells. A, Examples of chromatid breaks 3 h after 0.5 Gy γ-irradiation. The nascent chromatid breaks (as indicated by arrows) were identified by the chromatid gap larger than the width of the chromatid and the lack of telomere signals. The short arrows indicate the subtle terminal chromatid breaks. Telomeres were detected by red signals and DNA was stained blue by DAPI. B and C, Frequencies of chromatid breaks before or after γ-irradiation. Error bars represent standard deviations. For all time points analyzed, the frequencies of chromatid breaks in LMP1-expressing cells were significantly higher (*P*<0.05) than empty vector-infected cells.

Mitotic cells escaped from defective G2 checkpoint commonly exhibit chromatid-type aberrations due to inefficient DNA damage repair [19]. We therefore examined chromosome aberrations in metaphases of LMP1-expressing and vector-infected cells 2–8 h after 0.5 Gy γ-ray irradiation (Table S1). Control cells not irradiated with γ-ray were also examined. We used 4′,6-diamidino-2-phenylindole (DAPI) staining in combination with telomere fluorescence *in situ* hybridization to identify chromatid break points, as intact terminal chromatid ends would be protected by telomeres whereas unrepaired fresh breakpoints would be deprived of telomeres. Our analysis confirmed that the broken ends of all chromatid breaks detected were void of telomere signals, indicating nascent chromatid breaks (exemplified by the broken ends pointed by arrows in Figure 2A). With this technique, the subtle terminal chromatid breaks could be readily identified (indicated by short arrows in Figure 2A). In both HONE1 and NP460hTERT cell lines, no significant increase in the background frequencies of chromatid breaks (indicated by arrows in Figures 2B and 2C) as well as other chromosome aberrations was detected in LMP1-expressing cells (Table S1). Two to eight hours after 0.5 Gy γ-ray irradiation, the mitotic cells from both LMP1-expressing cell lines exhibited significantly higher frequencies of chromatid breaks than control empty vector-infected cells (*P*<0.05 for all analyzed time points) (Figures 2B and 2C). There was no significant increase in the frequencies of identifiable chromosomal-type aberrations, i.e., dicentrics, rings and double minutes (with both chromatids exhibiting the same break points) after irradiation in LMP1-expressing and empty vector-infected cells (Table S1), indicating that the chromatid breaks detected in the analyzed metaphases were initiated at G2 or late S phase. Irrespective of LMP1 expression, the frequencies of chromatid rearrangement after irradiation were rather low as compared with chromatid breaks (Table S1), suggesting that the chromosome repair through chromatid exchange in G2 phase was restrained.

The time course examination of the changes in the frequency of chromatid breaks from 2–8 h after irradiation (Figures 2B and 2C) revealed that the cells which entered mitosis at later time points after irradiation had fewer chromatid breaks, indicating that a longer G2 arrest facilitated repair of chromatid breaks. But LMP1-expressing cells persistently exhibited higher chromatid breaks compared to empty vector-infected control cells during the entire time course of analysis from 2 to 8 h after irradiation. Even when the mitotic index had recovered to pre-irradiation levels (see Figures 1A and 1B) at 8 h after irradiation, elevated chromatid breaks in LMP1-expressing cells could still be detected (Figures 2B and 2C). These results demonstrated that chromatid breaks were not completely repaired in the absence of G2 arrest after irradiation, which is consistent with a previously published report [19]. These results support that LMP1 expression suppresses the repair efficiency of chromatid breaks in G2 phase.

### LMP1 Impaired Chk1 Activation after γ-ray Irradiation

**Figure 3:**
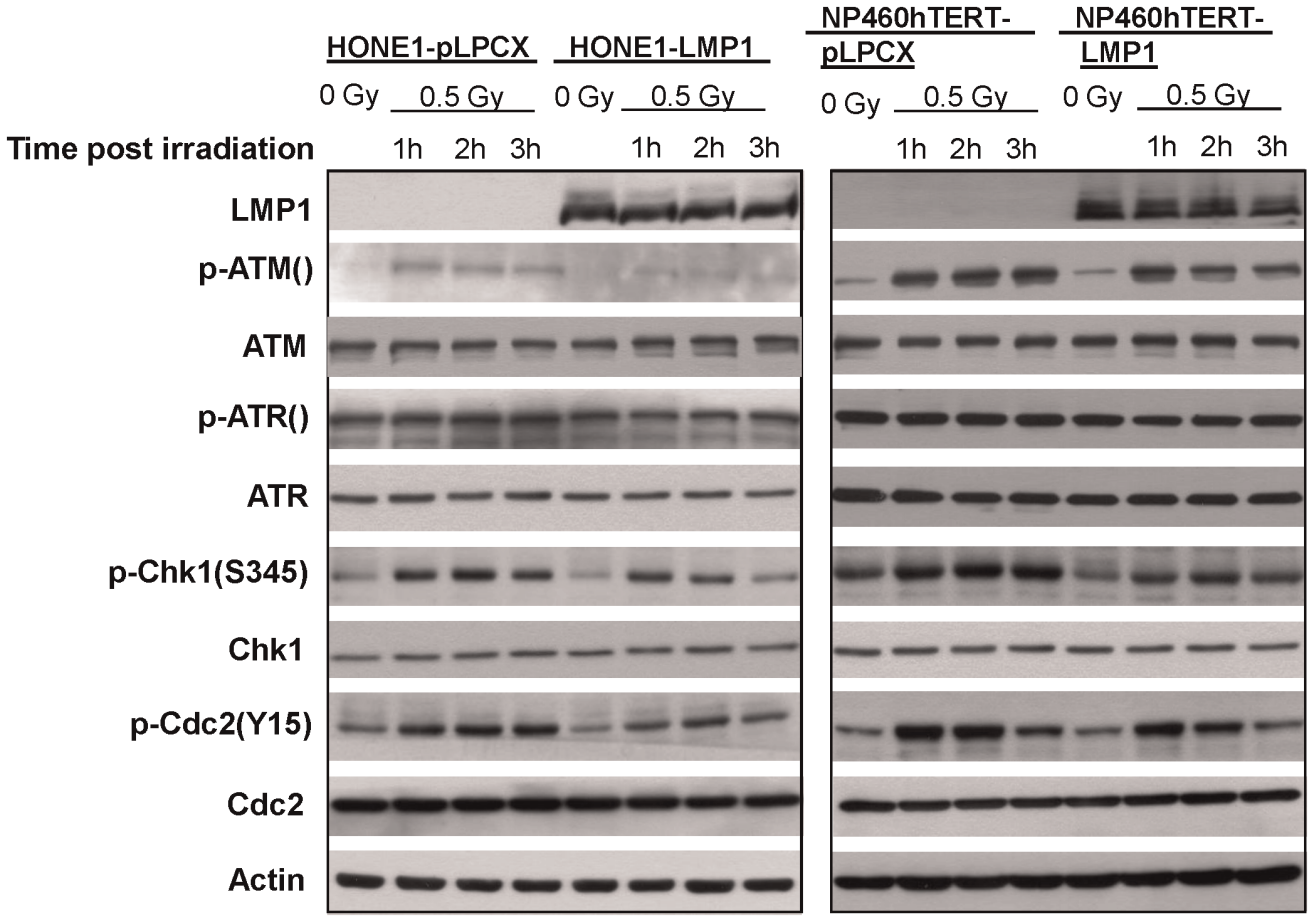
Western Blotting analysis in LMP1-expressing and control cells. Protein expression analysis by Western Blotting of various proteins related to Chk1 activation in cells without γ-irradiation or at different time points after 0.5 Gy γ-irradiation. Actin shows protein loading controls.

We next sought to understand the mechanism underlying the LMP1-induced G2 checkpoint defect in nasopharyngeal epithelial cells. It has been established that Chk1 activation plays an essential role in G2 checkpoint control [22], [23]. The ultimate target of Chk1 in G2 checkpoint is Cdc2-cyclin B complex. Chk1 is an effector protein kinase that maintains Cdc2 in an inhibitory state [23], [24], which is manifested by phosphorylation of Cdc2 on Tyr-15 and Thr-14. The inhibitory state of Cdc2 is crucial for preventing cell cycle transition from G2 to M phase. Phosphorylation of Chk1 on S345 is regarded as an indicator of Chk1 activation [25]. We therefore tested whether LMP1-induced defective G2 checkpoint involved defective Chk1 activation or not. Indeed, in both HONE1-LMP1 and NP460hTERT-LPM1 cell lines, the levels of phosphorylated Chk1(S345) were remarkably lower as compared with control cells 1–3 h after 0.5 Gy γ-irradiation (Figure 3). In agreement with the expected function of Chk1 in G2 checkpoint control, the inhibitory phosphorylation levels of Cdc2 on Tyr-15, p-Cdc2(Y15), in LMP1-expressing cells were also lower than those in control cells 1–3 h after γ-ray irradiation (Figure 3). The expression levels of total Chk1 and Cdc2 showed no significant differences between LMP1-expressing and empty vector-infected cells in response to γ-ray exposure.

We next checked the upstream regulators of Chk1. Phosphorylation of ATM and ATR has been shown to activate Chk1 and G2 checkpoint upon DNA damage [24]. Based on the deficient activation of Chk1 in LMP1-expressing cells, we therefore asked if stable LMP1 expression may interfere with the activation of ATM and/or ATR. As shown in Figure 3, ATM phosphorylation levels in LMP1-expressing cells were remarkably lower than empty vector-infected cells 1–3 h after irradiation, while no consistent differences were observed in the expression levels of total ATM, total ATR and phosphorylated ATR between LMP1-expressing and empty vector-infected cells. These results suggested that the impaired Chk1 activation in LMP1-expressing cells was associated with deficient ATM activation in response to DNA damage.

### Overexpression of Chk1 Improved G2 Checkpoint Function in LMP1-expressing Cells

**Figure 4:**
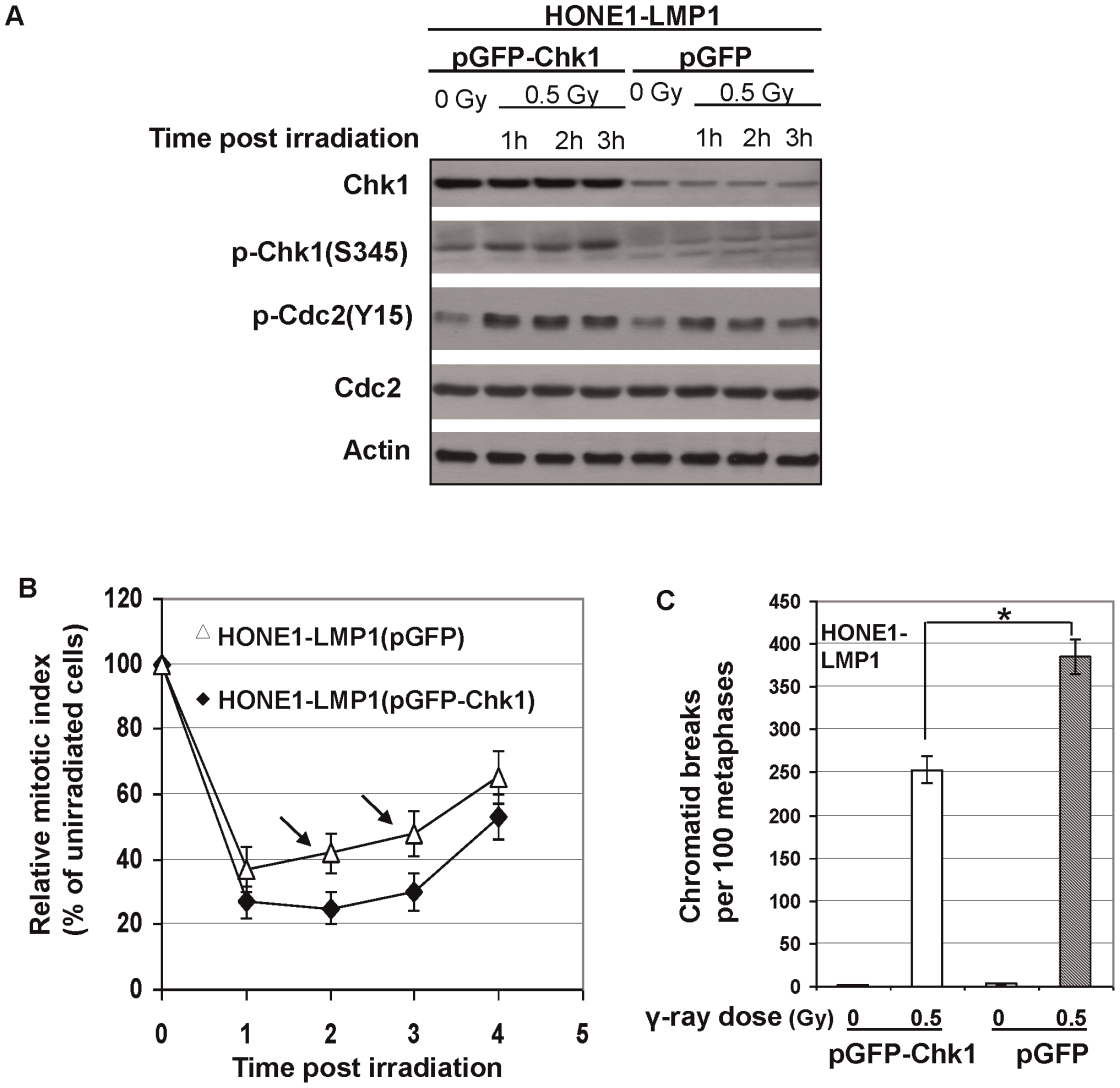
G2 checkpoint improvement in HONE1-LMP1 cells with ectopic Chk1 overexpression. A, Western Blotting analysis of Chk1, p-Chk1(S345), p-Cdc2(Y15) and Cdc2. Actin shows protein loading controls. B, Relative mitotic index at different time points after γ-ray irradiation. Error bars represent standard deviations. Arrows indicated the relative mitotic indices in HONE1-LMP1 cells transfected with control plasmids (pEGFP) significantly higher (*P*<0.05) than HONE1-LMP1 cells transfected with Chk1-expressing plasmids (pEGFP-Chk1). C, Frequencies of chromatid breaks before or after γ-irradiation. Error bars represent standard deviations. Stars indicated significant differences (*P*<0.05) between the indicated datum pairs.

To investigate whether impaired Chk1 activation in LMP1-expressing cells was truly responsible for the defective G2 checkpoint function in response to DNA damage, we transiently overexpressed Chk1 in HONE1-LMP1 cells to examine if defective G2 checkpoint function could be rescued. Western blotting analysis confirmed the successful overexpression of Chk1 in the cells (Figure 4A). Our results showed that overexpression of Chk1 enhanced the phosphorylation of Chk1 on S345 as compared with vector control cells with or without γ-ray irradiation, which also correlated with increased phosphorylation of Cdc2 on Y15 which inhibits G2 to M phase transition (Figure 4A). In response to irradiation, Chk1 overexpression overcame the ability of LMP1 to induce G2 checkpoint defect, as indicated by the decrease in relative mitotic index compared with control vector-transfected cells (Figure 4B). In line with these results, the frequency of chromatid breaks at 3 h after 0.5 Gy γ-irradiation in Chk1-overexpressing HONE1-LMP1 cells was also significantly lower than control cells (Figure 4C). These data together demonstrated that G2 checkpoint defect in LMP1-expressing cells could be rescued by ectopic enhancement of Chk1 function. This supports the idea that impaired Chk1 activation plays a crucial role in the induction of G2 checkpoint defect in LMP1-expressing cells.

## Discussion

We have shown, for the first time, that LMP1 encoded by NPC-derived EBV impaired G2 checkpoint through deficient activation of Chk1 in human nasopharyngeal epithelial cells. As a result, LMP1-expressing nasopharyngeal epithelial cells exhibited increased frequencies of unrepaired chromatid breaks in mitotic cells compared with control cells in response to γ-ray irradiation. Some of the broken chromatid fragments lacking centromeres may be lost from daughter cells during mitosis leading to loss of genetic materials, while some of the broken chromatids with centromeres may be propagated into daughter cells and become the source for further chromosome arrangements (mistaken repair). Dynamic generation of chromosome aberrations is the major form of genomic instability in cancer development [26]. Human cells are continuously exposed to various endogenous and exogenous genotoxic insults such as ionizing radiation, genotoxic chemicals, and byproducts of normal cellular metabolism that generate free radicals leading to DNA lesions. We therefore infer that LMP1 may contribute to genomic instability in EBV-infected nasopharyngeal epithelial cells under genotoxic insults.

In this study we mainly focused on chromosome aberrations in mitotic cells progressed from G2 cells that were exposed to ionizing radiation. Usually, the G2 phase of human cells *in vitro* lasts about 4 hours in the absence of irradiation [27]. The enhanced chromatid breaks in mitotic cells observed in this study in LMP1-expressing cells 2–4 h after irradiation were most likely stemmed from the breaks generated in earlier G2 phase and these breaks remain unpaired throughout the time course. In addition, we also extended the time points of chromosome aberration analysis to 6–8 h after γ-ray irradiation to obtain a better picture of time course changes in chromosome aberrations. For the later time points, we could not exclude the possibility that the aberrant metaphases detected were initiated at late S phase during γ-ray irradiation, which then progressed through G2 phase with incomplete repair of chromatid breaks to enter metaphase. Interestingly, even at the time when G2 arrest was no longer detected, i.e., 8 hours post irradiation, we could still detect enhanced chromatid breaks in LMP1-expressing cells as compared with empty vector-infected cells. It has been previously discovered that cells have a threshold of DNA damage to trigger G2 arrest [12], [19]. LMP1 expression clearly elevated this threshold, allowing more chromatid breaks to remain unrepaired when mitosis ensued. Our data in Figures 2 B and 2C showed that the differences between the frequencies of chromatid breaks in LMP1-positive and LMP1-negative cells at 8 h after irradiation were smaller than that at 2 h after irradiation. This may suggest that the LMP1 positive cells have a higher capacity of repairing chromatid breaks compared to LMP1 negative cells. In an earlier study, a similar trend of chromatid break repair rate was observed in G2 defective cells induced by inactivation of ATM [19]. The underlying mechanism is unclear at this stage. It remains to be determined if this phenomenon is related to LMP1 expression or a property of G2 checkpoint defective cells.

Our finding that LMP1 impairs G2 checkpoint in nasopharyngeal epithelial cells extends previous findings on the role of LMP1 in affecting DNA damage repair [28], [29]. ATM plays critical roles in both DNA damage repair and cell cycle checkpoint control. In B cells, the total protein levels of ATM and its phosphorylated form were found to be significantly down-regulated by LMP1 [29]. In the present study, we neither detected the decrease in total protein levels of ATM after LMP1 expression in nasopharyngeal epithelial cells in the absence of γ-ray irradiation, nor did we detect any significant change in total ATM protein levels after γ-ray irradiation. This is consistent with another previous report showing that ATM total protein levels were not correlated with LMP1 expression in NPC specimens and cell lines [30]. In this study, we did detect a deficient phosphorylation of ATM in LMP1-expressing nasopharyngeal epithelial cells 1–3 h after 0.5 Gy γ-ray irradiation, indicating impaired activation of ATM protein. Interestingly, another recent study reported that LMP1 expression in CNE1 and HNE2 cells, which were established from poorly differentiated nasopharyngeal squamous carcinomas, resulted in increased ATM expression; and downregulation of LMP1 expression could reduce the level of ATM expression, rendering the cells more sensitive to ionizing radiation [31]. The regulatory role of LMP1 on ATM appears to be dependent on cellular context. LMP1 may play distinct roles in NPC at different stages of development and tumorigenicity. The differential responses to LMP1 expression between B cells and nasopharyngeal epithelial cells are not surprising. Accumulating data demonstrate that B cells behave differently from epithelial cells after EBV infection and expression of EBV-encoded genes [1]. Meanwhile, it is worthwhile to note that in this study we specifically studied the functions of a Hong Kong-prevalent EBV-encoded LMP1 variant, 2117-LMP1, in nasopharyngeal epithelial cells, whereas an LMP1 cloned from B95-8 EBV [6] was used in the other study showing ATM down-regulation by LMP1 in B cells [29]. While B95-8-LMP1 is relevant to B cell malignancies, we reason that 2117-LMP1 might be more relevant to NPC development, in particular to the high-incidence areas of NPC, based on the finding that the EBV strain encoding 2117-LMP11 was present in the vast majority (86%) of NPC specimens in Hong Kong [16]. The mechanism for the defective ATM activation in 2117-LMP1-expressing nasopharyngeal epithelial cells remains unclear at this stage.

Since G2 checkpoint was the focus of this study, we checked the downstream targets of ATM activation involved in G2 checkpoint control. Impaired Chk1 activation as indicated by phosphorylation of Chk1 on S345 in response to γ-ray irradiation was found in our cell models expressing LMP1. As a downstream target of Chk1 activation, the inhibitory phosphorylation of Cdc2, the ultimate protein participating in controlling G2 to M phase transition, was also impaired. The ectopic overexpression of Chk1 in LMP1-exprssing cells enhanced Chk1 activation after γ-ray irradiation. This in turn resulted in the enhancement of inhibitory phosphorylation of Cdc2 and improvement of G2 checkpoint as well as decrease in γ-ray-induced chromatid breaks in metaphases after G2 release. Notably, the impaired phosphorylation, not the expression of total level of Chk1, was impaired in LMP1-expressing cells after irradiation. In this study, we have overexpressed Chk1 to rescue the defective Chk1 function which may not be identical to impaired Chk1 phosphorylation. Nonetheless, the ultimate purpose of this experiment was to restore the function of Chk1, which was reflected by the phosphorylation of Chk1 on S345, an indicator of the functional activation of Chk1 [25], in Chk1 overexpressing cells. Chk1 overexpression has been also used previously to restore G2 checkpoint function [32], [33]. Taken together, these results demonstrated the pivotal role of defective Chk1 function in G2 checkpoint deficiency in LMP1-expressing nasopharyngeal epithelial cells in response to DNA damage. Since Chk1 also functions in S phase checkpoint [24], the possible role of LMP1 in inducing defect in S phase checkpoint is under active investigation in our laboratory.

In summary, we have provided the first evidence that LMP1 enhances the formation of γ-ray-induced chromatid breaks in metaphases of human nasopharyngeal epithelial cells by impairing G2 checkpoint function. These unrepaired chromatid breaks may be lost from daughter cells or undergo chromosomal rearrangements after they are propagated into daughter cells after mitosis, generating further chromosome aberrations. This study suggests that LMP1 expression could induce genomic instability in nasopharyngeal epithelial cells under genotoxic stress, which is continuously faced by human cells. Further studies on the impact of interaction between genotoxic microenvironment and EBV infection on NPC pathogenesis are warranted.

## Materials and Methods

### Cell Culture and Irradiation

Immortalized NP460hTERT cell line which was previously established at this laboratory [15] and its LMP1-expressing counterpart were cultured using 1∶1 mixture of growth factor-supplemented Defined Keratinocyte-Serum Free Medium (GIBCO, Invitrogen, Carlsbad, CA) and EpiLife medium (Cascade Biologics, Invitrogen, Carlsbad, CA). NPC cell line HONE1 [14], a kind gift from Prof. Ronald Glaser (The Ohio State University Medical Center, Columbus, OH), was cultured using RPMI 1640 medium (Sigma, St. Louis, MO) supplemented with 10% fetal bovine serum (GIBCO). The culture media were refreshed 1 d before irradiation, which was carried out when cell confluence reached about 80%. Ionizing γ-ray irradiation was carried out at a dose rate of 10 Gy/min in a GammaCell 220 irradiator containing a ^137^Cs radiation source (Atomic Energy of Canada Ltd.).

### Retroviral Infection

HONE1 [14] and NP460-hTERT [15] cells were infected with retroviral vector pLPCX-2117LMP1 generated at this laboratory or empty vector pLPCX (BD Biosciences, San Jose, CA).) using 4 µg/ml polybrene (Sigma-Aldrich). Infectious retroviruses were prepared as reported [34]. Three days after retroviral infection, the immortalized and NPC cell lines were selected with 0.5 and 1 µg/ml puromycin, respectively, for 6 days. The puromycin-resistant cells were pooled for experiments.

### Plasmids Transfection

The pEGFP-Chk1 plasmids were a kind gift from Prof. Helen Piwnica-Worms, Washington University School of Medicine, U.S.A. [25]. The control pEGFP plasmids were purchased from Clontech (Mountain View, CA). Plasmid transfection into HONE1-LMP1 cells were carried out using Fugene HD transfection reagent (Roche) according to the manufacturer’s instruction.

### Chromosome Spreading for Mitotic Index Analysis

Cells were harvested without addition of any microtubule inhibitors. Chromosome spreading was performed using protocols previously reported [35]. Cells with distinguishable individual chromosome spreads were identified as mitotic cells. For each experiment point, at least 5,000 cells were counted.

### Chromatid Break Analysis and Telomere Fluorescence *in Situ* Hybridization (FISH)

The chromatid gap larger than a width of the chromatid was scored as a chromatid break. We used telomere FISH to confirm the chromatid breaks because intact terminal ends would carry telomere signals while fresh broken ends would lack telomere signals. Cy3-conjugated peptide nucleic acid (PNA) telomere probes were purchased from Dako (Denmark). Telomere FISH and DAPI staining were performed as reported [36]. One hundred metaphases were analyzed for each experiment point.

### CENP-F Immunofluorescence

Immunofluorescence was performed according methods described [37]. Primary rabbit antibodies against CENP-F (ab5) (Abcam, UK) were applied at a dilution of 1∶500. Anti-rabbit secondary antibodies conjugated with rhodamine (Molecular Probes, Oregon) applied at a dilution of 1∶1000 were used for fluorescence staining. Cells were counterstained with DAPI. Immunofluorescence images were acquired using a Leica fluorescence microscope equipped with a CCD camera, which was controlled by a computer using SPOT software (Leica).

### Western Blotting

Twelve microgram protein was separated by 7.5–10% SDS-PAGE and blots were prepared on a polyvinylidene fluoride membrane (Amersham). The primary antibodies were from the following sources: anti-Actin and anti-Chk1 (G-4) from Santa Cruz Biotechnology (Santa Cruz, CA), anti-LMP1 (S12) from BD Pharmingen (San Jose, CA), anti-ATM from Millipore (Billerica, MA), anti-pATM(S1981) from Epitomics (Burlingame, CA), anti-ATR, anti-pATR(S428), anti-Cdc2, and anti-pCdc2(Y15) from Cell Signaling Technology (Beverly, MA). The membrane was probed with secondary antibody against peroxidase-conjugated mouse, rabbit, or goat IgG (Cell Signaling Technology), and the blots were visualized by the enhanced chemiluminescence Western blotting system (Amersham).

### Statistical Analysis

The two-tailed T-test was used to examine the statistical differences. *P* values <0.05 were regarded as significant. In all bar graphs, error bars represent standard deviations. For calculation of standard deviation of relative value R  =  A ÷ B (for relative mitotic index), the following formula was used:

(σR/R)^2^ =  (σA/A)^2^+ (σB/B)^2^ where σR/R, σA/A and σB/B are relative standard deviation of R, A and B, respectively.

## Supporting Information

Figure S1 **CENP-F staining of mitotic cells**. Mitotic cells could be identified by the discrete CENP-F staining as well as condensed chromatin.
(PPT)

Table S1 **Chromosome aberrations in LMP1-expressing and control cell lines before and after γ-ray irradiation (IR).** Chromosome aberrations were analyzed in 100 metaphases using DAPI staining in combination with telomere FISH.
(DOC)
