# Supplementary figures and images for: Epstein-Barr Virus-Encoded Latent Membrane Protein 1 Impairs G2 Checkpoint in Human Nasopharyngeal Epithelial Cells through Defective Chk1 Activation
Source: PLoS One. 2012 Jun 25;7(6):e39095. doi: 10.1371/journal.pone.0039095 (PMC3382577; doi:10.1371/journal.pone.0039095)

## Slide 1
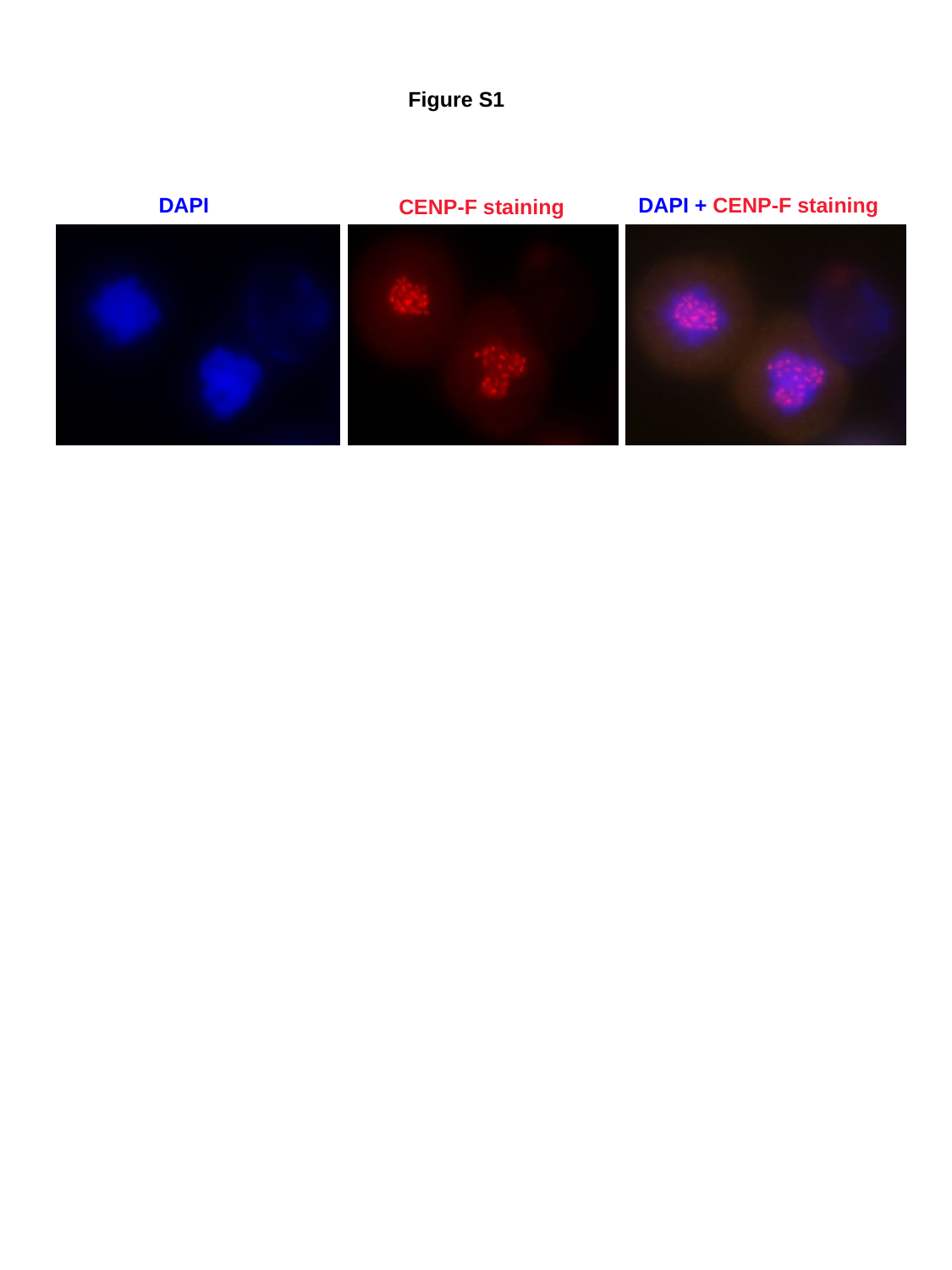

Figure S1
DAPI
DAPI + CENP-F staining
CENP-F staining

Supplement: Figure S1 — CENP-F staining of mitotic cells. Mitotic cells could be identified by the discrete CENP-F staining as well as condensed chromatin. (PPT) [file pone.0039095.s001.ppt]
