# Supplementary material for: Epstein-Barr Virus-Encoded Latent Membrane Protein 1 Impairs G2 Checkpoint in Human Nasopharyngeal Epithelial Cells through Defective Chk1 Activation
Source: PLoS One. 2012 Jun 25;7(6):e39095. doi: 10.1371/journal.pone.0039095 (PMC3382577; doi:10.1371/journal.pone.0039095)
Supplement: Table S1 — Chromosome aberrations in LMP1-expressing and control cell lines before and after γ-ray irradiation (IR). Chromosome aberrations were analyzed in 100 metaphases using DAPI staining in combination with telomere FISH. (DOC) [file pone.0039095.s002.doc]

**Table S1**

| Cell line | Non-clonal chromosome aberrations | | | | | | | | | | | | | | | | | | | |
| --- | --- | --- | --- | --- | --- | --- | --- | --- | --- | --- | --- | --- | --- | --- | --- | --- | --- | --- | --- | --- |
|  | Chromatid breaks | | | | | Chromatid arrangements | | | | | Dicentrics and rings | | | | | Double minutes | | | | |
|  | Pre-IR | Time post-IR (h) | | | | Pre-IR | Time post-IR (h) | | | | Pre-IR | Time post-IR (h) | | | | Pre-IR | Time post-IR (h) | | | |
|  |  | 2 | 3 | 5 | 8 |  | 2 | 3 | 5 | 8 |  | 2 | 3 | 5 | 8 |  | 2 | 3 | 5 | 8 |
| HONE1-MP1 | 2 | 532 | 404 | 300 | 248 | 0 | 4 | 5 | 6 | 5 | 3 | 2 | 3 | 4 | 3 | 7 | 10 | 7 | 6 | 7 |
| HONE1-pLPCX | 2 | 335 | 275 | 223 | 201 | 0 | 4 | 6 | 7 | 6 | 2 | 3 | 2 | 4 | 4 | 5 | 7 | 5 | 6 | 6 |
| NP460hTERT-LMP1 | 0 | 356 | 267 | 198 | 163 | 0 | 2 | 4 | 4 | 6 | 1 | 2 | 1 | 3 | 3 | 0 | 2 | 3 | 2 | 3 |
| NP460hTERT-pLPCX | 0 | 248 | 190 | 153 | 125 | 0 | 2 | 3 | 5 | 4 | 0 | 2 | 2 | 4 | 2 | 0 | 3 | 2 | 3 | 4 |
